# Dye-Free Porcine Model of Experimental Branch Retinal Vein Occlusion: A Suitable Approach for Retinal Proteomics

**DOI:** 10.1155/2015/839137

**Published:** 2015-05-03

**Authors:** Lasse Jørgensen Cehofski, Anders Kruse, Benedict Kjærgaard, Allan Stensballe, Bent Honoré, Henrik Vorum

**Affiliations:** ^1^Department of Ophthalmology, Aalborg University Hospital, Hobrovej 18-22, 9000 Aalborg, Denmark; ^2^Biomedical Research Laboratory, Aalborg University Hospital, Ladegårdsgade 3, 9000 Aalborg, Denmark; ^3^Department of Heart and Lung Surgery, Center for Cardiovascular Research, Aalborg University Hospital, Hobrovej 18-22, 9000 Aalborg, Denmark; ^4^Department of Health Science and Technology, Aalborg University, Fredrik Bajers Vej 3, Building B, 9220 Aalborg, Denmark; ^5^Department of Biomedicine, Aarhus University, Ole Worms Allé 3, Building 1182, Room 024, 8000 Aarhus C, Denmark

## Abstract

Branch retinal vein occlusion induces complex biological processes in the retina that are generated by a multitude of interacting proteins. These proteins and their posttranslational modifications can effectively be studied using modern proteomic techniques. However, no method for studying large-scale protein changes following branch retinal vein occlusion has been available until now. Obtainment of retinal tissue exposed to branch retinal vein occlusion is only available through experimental animal models. Traditional models of experimental branch retinal vein occlusion require the use of Rose Bengal dye combined with argon laser photocoagulation. The use of Rose Bengal dye is problematic in proteomic studies as the dye can induce multiple protein modifications when irradiated. This paper presents a novel technique for proteomic analysis of porcine retinal tissue with branch retinal vein occlusion combining a dye-free experimental model with label-free liquid chromatography mass spectrometry based proteomics.

## 1. Introduction

Branch retinal vein occlusion (BRVO) is the second most frequent retinal vascular disease after diabetic retinopathy [1]. The disease is a common cause of visual loss in middle aged and elderly people [2]. The predominant pathogenetic mechanism for BRVO is thought to be compression of the retinal vein by a thickened retinal artery [2] that occurs at a site of arteriovenous crossing [3]. This mechanical narrowing of the venous lumen results in widespread flame-shaped haemorrhages, axonal congestion, dilated tortuous veins, ischaemia, and oedema upstream of the occlusion [2, 4].

In retinal tissue BRVO induces complex biological processes that are driven by a multitude of interacting proteins. For example, the VEGF-VEGF2 signalling pathway has been shown to have an essential role in BRVO [5]. Large-scale protein changes following BRVO can effectively be studied using proteomic technologies to bring new insights into pathological processes and signaling pathway alterations that occur in the disease [6–8]. However, no method for analyzing large-scale protein changes following BRVO has been established.

From human eyes with BRVO, retinal tissue samples are generally only obtainable at postmortem [9, 10] which makes experimental models of large animals better suited for studying retinal protein changes using proteomics [11]. Porcine retinas are highly suitable for experimental studies of BRVO. Like the human retina the porcine retina is fully vascularised which is an advantage if hypoperfusion and ischemia develop [12]. Furthermore, porcine and human eyes are largely identical in size [12]. Therefore, porcine eyes may render a sufficient amount of retinal tissue for proteomic studies. In addition, the size of porcine eyes makes them suitable for validation with fluorescein angiography and fundus photography [13].

Existing experimental models of BRVO are predominantly based on photothrombosis [14–16]. With photothrombosis the animal receives an intravenous injection of Rose Bengal dye followed by application of argon green laser burns directly on the branch vein which creates an intravascular thrombus [14, 15]. The peak absorption of Rose Bengal is close to the wavelength of argon laser which makes it possible to use less laser energy [17].

When exposed to visible light Rose Bengal can induce posttranslational modifications. Therefore, experimental BRVO is best induced without the use of Rose Bengal if the retinal tissue is intended for proteomic experiments. In this paper we introduce a novel method that allows for large-scale protein identification of more than 2000 proteins in porcine retinal tissue exposed to experimental BRVO by combining a dye-free experimental model with label-free liquid chromatography mass spectrometry based proteomics.

## 2. Materials and Methods

The study was approved by the Danish Animal Experiments Inspectorate (permission number 2013-15-2934-00775). A Danish Landrace pig, approximately 30 kg, was anesthetized with an intramuscular injection of Zoletil (ketamine 6.25 mg/mL, tiletamine 6.25 mg/mL, zolazepam 6.25 mg/mL, butorphanol 1.25 mg/mL, and xylain 6.25/mL).

### 2.1. Experimental Branch Retinal Vein Occlusion

The eyes were anesthetized with Oxybuprocaine Hydro 0.4% (Bausch & Lomb) and Tetracaine 1% (Bausch & Lomb) followed by dilatation with Tropicamide 0.5% (Mydriacyl; Bausch & Lomb) and Phenylephrine 10% (Metaoxidrin; Bausch & Lomb). To prevent the corneal surface from becoming dry and compromise the view of the retina Systane Ultra eye drops (Polyethylene Glycol 400, Propylene Glycol; Alcon, Copenhagen, Denmark) were applied every 5 minutes.

**Figure 1:**
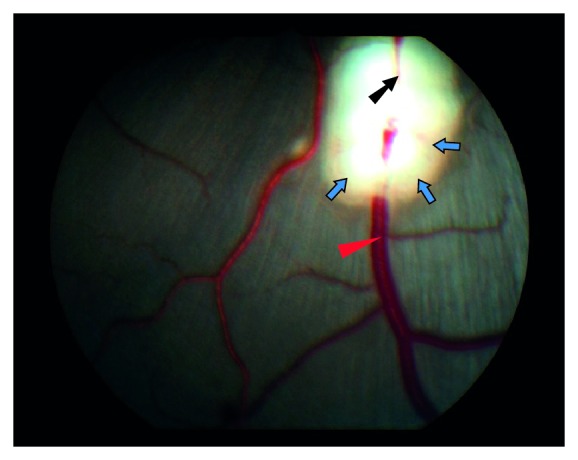
Dilation of vein due to downstream occlusion. The dilated section of the vein is marked with red triangle. The laser patch is marked with blue arrow. The site of occlusion is marked with black arrow.

BRVO was induced using a standard argon laser (532 nm) that was given by indirect ophthalmoscopy. Initially laser burns were applied around an inferior branch vein to narrow the vessel. The following laser application was given directly on the vein until stagnation of the venous blood flow occurred. After 37 laser applications with a power of 400 mW and a duration of 550 ms the venous blood stagnated in the vein (Figure 1). Peripheral flame-shaped hemorrhages appeared within 30 minutes after the occlusion had been induced.

In the inferior retina of the left eye that served as a control, laser was applied in a similar area that was free of vessels. A validating fluorescein angiography was conducted nine days after induction of the BRVO to confirm successful occlusion. By day 15 the pig was examined with ophthalmoscopic examination and fundus photography followed by enucleation of the eyes that were stored at −80°C until preparation for proteomic analysis. Euthanasia of the pig followed immediately after enucleation. The right eye with BRVO was used for proteomic analysis.

### 2.2. Sample Preparation for Proteomic Analysis

The porcine eye was thawed on ice. A circumferential incision was made around the iris 2 mm posteriorly to the limbus to remove the cornea and the iris. The lens and the vitreous body were carefully lifted and the retina was peeled from the eye cup and placed in an Eppendorf tube with five 2.3 mm chromium balls. The Eppendorf tube was placed on a Vortex mixer for approximately 30 seconds to homogenize the sample. A volume of 500 *μ*L cold PBS was added to the Eppendorf tube followed by another 30 seconds of homogenization on the Vortex mixer. A volume of 50 *μ*L of the homogenate was placed in a bead beading tube containing 500 *μ*L 5% sodium deoxycholate (NaDOC), pH 8.5, and homogenized by bead beading with a Precellys 24 homogenizer (Bertin Technologies, Rockville, MD, USA) at 6,000 rpm for 20 sec per round. The samples were kept on ice between bead beading runs. The retinal sample was placed in a YM-10 Spin Filter (Millipore) and buffer exchange to digestion buffer (1% NaDOC in 0.1 M TEAB; pH 7.8) was performed. This was followed by reduction at 37°C using 12 mM TCEP for 30 minutes and alkylation with 40 mM iodoacetamide for 30 minutes followed by two times buffer exchange by centrifugation at 14.400 g into 0.5% NaDOC in 0.1 M TEAB; pH 7.8. For digestion two ug trypsin (modified, Promega) was added for incubation overnight. The filtrate was recovered and sodium deoxycholate was precipitated by adding 5% formic acid followed by recovery of the soluble peptides. An internal standard of 20 fm/uL BSA digest (Waters Massprep) was added and the retinal sample was analyzed by nano-UPLC MS.

### 2.3. Liquid Chromatography Tandem Mass Spectrometry Data Acquisition

A sample volume of 5 *μ*L was transferred onto a Dionex RSLC nano-UPLC system connected to a Quadrupole Orbitrap (Q Exactive Plus) mass spectrometer that was equipped with a NanoSpray Flex ion source (Thermo Scientific, Bremen, Germany). The sample was loaded onto a trapping column (Acclaim PepMap100 C18, 5 *μ*m column from Thermo Scientific) at a flow rate of 8 *μ*L per min. Peptide separation was conducted at a nanoflow of 300 nL per min on the analytical column (50 cm Acclaim PepMap RSLC). The nanoelectrospray was conducted using a Picotip “Silicatip” emitter from New Objectives. Formic acid 0.1% (buffer A) and 99.9% acetonitrile with formic acid 0.1% (buffer B) were used as buffers. The applied gradient ranged between 10% and 30% over 230 minutes.

Operating in data-dependent acquisition mode a full MS scan in the mass range of 350 to 1400* m/z* was obtained at a resolution of 70,000 with an AGC target of 10^6^ and maximum fill time set to 100 ms. The contaminant ion at 391.28429* m/z* was used for instrument lock mass correction. Up to 12 MS/MS acquisitions on abundant peptide precursor ions were triggered per cycle. The MS/MS scans were acquired with a dynamic mass range at a resolution of 17,500 and with an AGC target of 5 × 10^5^ and max fill time of 50 ms. A quadrupole isolation window of 2.0* m/z* was used for isolation of the precursor ions that were fragmented in the HCD trap with a normalised collision energy set to 30. An underfill ratio at 1.0% with intensity threshold at 1.0 × 10^5^ was used. Apex triggering was set to 3 s–10 s and dynamic exclusion was set to 30 s. The sample was analyzed in three technical replicates.

### 2.4. Protein Identification and Bioinformatics

Data obtained by mass spectrometry were searched against a pig isoform database optimized from Uniprot using MaxQuant (version 1.5.0.22). The label-free algorithm was activated in MaxQuant. The peptide and protein false discovery rates were set to 1%. A two-tailed, heteroscedastic *t*-test was used to calculate *p* values.

Identified proteins were uploaded onto Perseus (version 1.5.0.9). Technical replicates were averaged by mean and proteins identified by less than two unique peptides were removed from the dataset. Perseus was used to search for proteins that were identified from peptides that were found to be part of a protein derived from the reversed part of the decoy database. These proteins were also removed from the dataset. All identified proteins were exported to Excel (Microsoft Office Standard 2007). Remaining keratins in the dataset were removed. Proteins with no assigned gene name in the identification were searched for on http://www.uniprot.com/ and gene names corresponding to the identified proteins were added.

A bioinformatic analysis was conducted using GeneCodis3 [18–20]. Gene names corresponding to the identified proteins were entered in GeneCodis3 to conduct Gene Ontology cellular component analysis.* Homo sapiens* was selected as organism to ensure that all gene names were recognized by GeneGodis3. *p* values were obtained by hypergeometric analysis corrected by a false discovery rate method.

## 3. Results and Discussion

**Figure 2:**
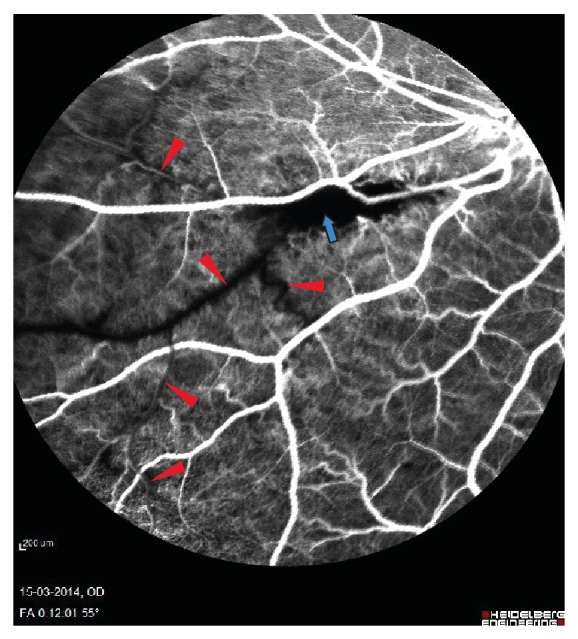
Delayed filling of occluded vein. The delayed filling is marked with red triangle. The site of occlusion is marked with blue arrow.

**Figure 3:**
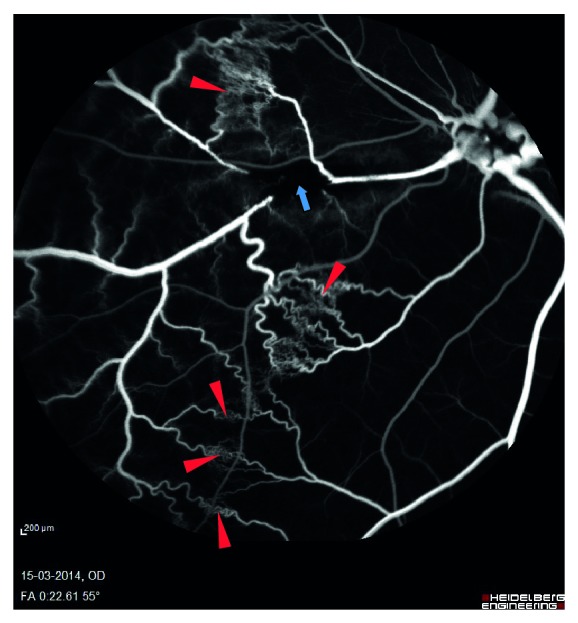
Shunting of blood for drainage by adjacent veins. Shunting of blood to adjacent veins is marked with red triangle. The site of occlusion is marked with blue arrow.

**Figure 4:**
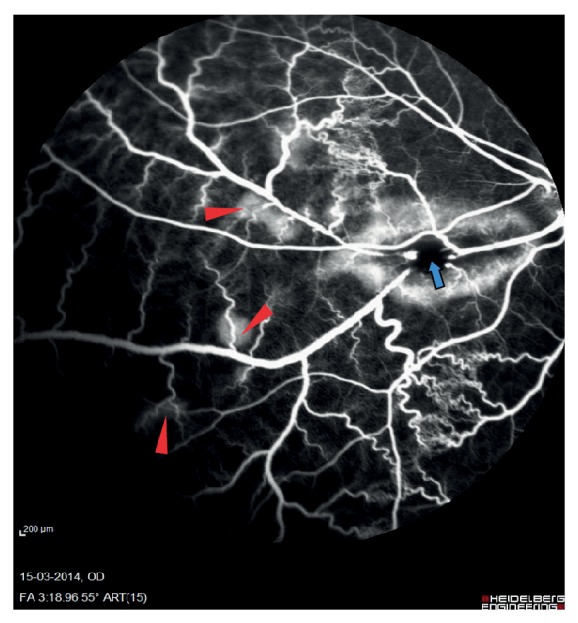
Peripheral haemorrhages. Peripheral haemorrhages appearing upstream to the occlusion are marked with red triangle. The site of occlusion is marked with blue arrow.

Fluorescein angiography nine days after experimental BRVO showed delayed venous filling of the occluded vein (Figure 2). Drainage of venous blood by adjacent veins (Figure 3) was observed in the angiography after approximately 12–14 seconds and peripheral haemorrhages (Figure 4) appeared after 30–40 seconds. The vein remained occluded for the entire duration of the angiography that lasted close to 12 minutes. Ophthalmoscopic examination by day 15 showed the retinal vein to be entirely occluded. Peripheral haemorrhages had been absorbed and no retinal edema was observed.

After filtering by the criteria described above the proteomic analysis provided identification of 2686 proteins in the retina that was exposed to BRVO. Gene Ontology analysis showed that a large variety of proteins were identified in the retinal sample (Table 1).

### 3.1. Dye-Free Photothrombosis versus Photothrombosis with Administration of Rose Bengal

Donati et al. [16] used a Rose Bengal concentration of 10 mg/kg that was injected intravenously in minipigs of 10 to 12 kg. BRVO was induced by applying argon green laser (wavelength 514 nm) on a branch vein close to the optic nerve head using an energy intensity of 250 mW, a pulse duration of 0.2 seconds, and a spot diameter of 500 *μ*m [16]. In our experiment without Rose Bengal the retinal vein remained unaffected when exposed to laser applications at 250 mW suggesting that the use of Rose Bengal reduces the amount of energy that is needed for BRVO to occur.

In a study on porcine eyes by McAllister et al. [15] an intravenous injection of 10 mg/kg of Rose Bengal was injected via an ear vein and BRVO was induced with an argon green laser (wavelength 514 nm) applied via a slit-lamp using the central position of a 3-mirror contact lens. With a spot size of 125 *μ*m, power of 150 mW, and duration of 1 second repeated laser burns (approximately 4-5) were applied directly to the inferior branch retinal vein until stagnation of the retinal blood flow was observed [15]. Though a duration of 1 second per laser application is a long exposure time, our experiment may indicate that a larger amount of laser applications is necessary to induce BRVO without Rose Bengal dye. Few laser applications may be an advantage in studies in which creation of the venous thrombus with minimal damage to the retinal vein is important.

Alternatively, BRVO can be induced in a porcine retina by establishing three sclerotomies in the porcine eye as in a three-port pars plana vitrectomy. By slowly lowering the diathermy probe toward the point of diathermia BRVO is induced by touching the vein with the diathermy probe for 6 seconds. No vitrectomy is performed [13]. However, this model requires access to surgical facilities and may affect the proteome of the vitreoretinal interface as the diathermy probe is in direct contact with the vitreous body and the retina.

### 3.2. Protein Modifications Induced by Rose Bengal

When exposed to visible light and oxygen, Rose Bengal produces singlet oxygen and reactive oxidative species, which can induce a number of protein modifications and interactions that may potentially affect the outcome of the proteomic study conducted [21]. Singlet oxygen (^1^O_2_) is known to oxidize the free amino acids Trp, Tyr, His, Cys, and Met resulting in alterations to protein structure and function [22–24]. These ^1^O_2_-mediated changes include oxidation of side chains, dimerization, aggregation, unfolding, conformational changes, and alterations in cellular handling and turnover [24]. In a study by Rahmanto et al. the thioredoxin reductase and glutathione peroxidase as purified proteins and as cell lysates were exposed to Rose Bengal and visible light which resulted in photolysis induced reductions in the activity of the enzymes [22].

Menon et al. [25] demonstrated that irradiation of retinal pigment epithelium (RPE) cells with visible light in the presence of Rose Bengal dye induces RPE cell lysis in vitro [25]. Rose Bengal has been shown to bind to albumin [26] which makes the dye likely to be distributed to several retinal layers when the blood-retinal barrier is disrupted due to BRVO. Furthermore, Rose Bengal has been shown to induce secondary and tertiary structure alternations of bovine *α*-crystallin in the presence of visible light [27].

### 3.3. Laser-Induced Effects on Protein Expression

Laser photocoagulation may potentially affect the retinal proteome. For example, in a proteome study by Quin et al. [28] laser photocoagulation was found to induce a downregulation of Wnt-5 beta and calretinin in Dark Agouti rats with streptozotocin-induced diabetes mellitus.

To measure the proteome change caused by BRVO and avoid the measurement of protein changes caused by laser treatment it is essential to create an adequate control with an area of laser burns that is as identical as possible to the eye with BRVO. The laser exposure in the eye with BRVO should be sufficient for the BRVO to be induced, but unnecessary laser should be avoided.

## 4. Conclusion

In this paper we describe a novel optimized method for proteomic analysis of retinal tissue exposed to experimental BRVO by combining a dye-free photocoagulation based porcine model with liquid chromatography tandem mass spectrometry. Dye-free photocoagulation resulted in successful induction of BRVO. The following proteomic analysis of the retinal tissue resulted in identification of 2686 proteins of various cellular compartments. For proteomic analysis of retinal tissue BRVO is best conducted without the use of Rose Bengal to avoid the multiple protein modifications that are induced when the dye is irradiated. However, induction of BRVO without Rose Bengal dye may require a larger number of laser applications and a higher level of laser energy. Therefore, creation of an adequate control with an area of laser burns that is similar to the eye with BRVO is essential to avoid measuring laser-induced proteome changes.

## Figures and Tables

**Table 1:** Gene Ontology cellular compartment analysis.

Annotations	Proteins	NGR	Hyp	Hyp^∗^
GO:0005737: cytoplasm	1106	5302	1.8 × 10^−289^	1.3 × 10^−286^
GO:0005634: nucleus	803	5441	1.2 × 10^−101^	2.14 × 10^−99^
GO:0005829: cytosol	633	2146	4.4 × 10^−234^	1.6 × 10^−231^
GO:0016020: membrane	551	4065	1.2 × 10^−52^	9.2 × 10^−51^
GO:0005886: plasma membrane	472	3575	3.1 × 10^−41^	1.4 × 10^−39^
GO:0005739: mitochondrion	453	1394	1.8 × 10^−181^	4.3 × 10^−179^
GO:0016021: integral to membrane	405	4400	6.1 × 10^−8^	5.2 × 10^−7^
GO:0005730: nucleolus	320	1461	6.2 × 10^−76^	8.8 × 10^−74^
GO:0005622: intracellular	230	1962	8.8 × 10^−14^	1.6 × 10^−12^
GO:0005783: endoplasmic reticulum	222	1000	2.8 × 10^−53^	2.5 × 10^−51^
GO:0005654: nucleoplasm	215	891	4.3 × 10^−58^	4.36 × 10^−56^
GO:0005856: cytoskeleton	187	846	1.4 × 10^−44^	8.4 × 10^−43^
GO:0005794: Golgi apparatus	174	958	3.5 × 10^−30^	1.2 × 10^−28^
GO:0005576: extracellular region	170	1913	0.003	0.008
GO:0005789: endoplasmic reticulum membrane	140	642	7.3 × 10^−33^	2.7 × 10^−31^
GO:0005625: soluble fraction	120	390	4.1 × 10^−44^	2.2 × 10^−42^
GO:0048471: perinuclear region of cytoplasm	115	417	3.3 × 10^−37^	1.4 × 10^−35^
GO:0005743: mitochondrial inner membrane	113	308	2.8 × 10^−50^	2.0 × 10^−48^
GO:0005759: mitochondrial matrix	104	201	3.3 × 10^−64^	3.9 × 10^−62^
GO:0005624: membrane fraction	98	523	2.0 × 10^−18^	5.0 × 10^−42^
GO:0005615: extracellular space	96	784	2.5 × 10^−7^	2.1 × 10^−6^
GO:0005887: integral to plasma membrane	94	1016	0.008	0.02
GO:0030054: cell junction	85	475	5.9 × 10^−15^	1.1 × 10^−13^
GO:0005840: ribosome	78	174	1.5 × 10^−42^	7.1 × 10^−41^
GO:0000139: Golgi membrane	75	420	2.6 × 10^−13^	4.4 × 10^−12^
GO:0045202: synapse	70	288	8.86 × 10^−20^	2.4 × 10^−18^
GO:0030529: ribonucleoprotein complex	69	132	2.2 × 10^−43^	1.1 × 10^−41^
GO:0031410: cytoplasmic vesicle	67	287	5.2 × 10^−18^	1.3 × 10^−16^
GO:0005792: microsome	64	279	7.3 × 10^−17^	1.6 × 10^−15^
GO:0005813: centrosome	63	323	3.7 × 10^−13^	6.1 × 10^−12^
GO:0042470: melanosome	59	89	1.1 × 10^−45^	7.0 × 10^−44^
GO:0005874: microtubule	59	262	2.8 × 10^−15^	5.7 × 10^−14^
GO:0009986: cell surface	57	297	9.6 × 10^−12^	1.3 × 10^−10^
GO:0043231: intracellular membrane-bounded organelle	56	269	4.7 × 10^−13^	7.4 × 10^−12^
GO:0015629: actin cytoskeleton	54	180	5.16 × 10^−20^	1.52 × 10^−18^
GO:0043234: protein complex	53	217	2.0 × 10^−15^	4.0 × 10^−14^
GO:0043025: neuronal cell body	48	217	2.1 × 10^−12^	3.2 × 10^−11^
GO:0005694: chromosome	48	265	3.0 × 10^−9^	3.3 × 10^−8^
GO:0071013: catalytic step 2 spliceosome	47	79	1.8 × 10^−33^	7.1 × 10^−32^
GO:0016607: nuclear speck	46	134	6.8 × 10^−20^	1.9 × 10^−18^
GO:0005768: endosome	45	280	3.5 × 10^−7^	2.6 × 10^−6^
GO:0030424: axon	43	173	4.6 × 10^−13^	7.3 × 10^−12^
GO:0030425: dendrite	42	182	1.2 × 10^−11^	1.5 × 10^−20^
GO:0005764: lysosome	40	179	1.0 × 10^−10^	1.2 × 10^−9^
GO:0019717: synaptosome	39	111	1.7 × 10^−17^	4.1 × 10^−16^
GO:0042995: cell projection	39	158	6.9 × 10^−12^	9.8 × 10^−11^
GO:0022625: cytosolic large ribosomal subunit	38	52	1.9 × 10^−32^	6.9 × 10^−31^
GO:0005741: mitochondrial outer membrane	37	102	3.4 × 10^−17^	7.8 × 10^−16^
GO:0031012: extracellular matrix	37	146	1.0 × 10^−11^	1.3 × 10^−11^
GO:0000502: proteasome complex	36	60	3.8 × 10^−26^	1.2 × 10^−24^
GO:0016323: basolateral plasma membrane	35	129	4.4 × 10^−12^	6.4 × 10^−11^
GO:0005681: spliceosomal complex	34	75	1.6 × 10^−19^	4.1 × 10^−18^
GO:0016324: apical plasma membrane	32	196	1.2 × 10^−5^	6.4 × 10^−5^
GO:0005938: cell cortex	31	106	8.7 × 10^−12^	1.2 × 10^−10^
GO:0005635: nuclear envelope	31	107	1.1 × 10^−11^	1.5 × 10^−10^
GO:0031965: nuclear membrane	30	133	1.7 × 10^−8^	1.6 × 10^−7^
GO:0005911: cell-cell junction	29	93	6.9 × 10^−12^	9.6 × 10^−11^
GO:0005788: endoplasmic reticulum lumen	29	106	2.4 × 10^−10^	2.8 × 10^−9^
GO:0005925: focal adhesion	28	116	1.0 × 10^−8^	1.1 × 10^−7^
GO:0030496: midbody	27	73	3.6 × 10^−13^	6.0 × 10^−12^
GO:0005769: early endosome	27	122	1.3 × 10^−7^	1.1 × 10^−6^
GO:0022627: cytosolic small ribosomal subunit	26	36	2.2 × 10^−22^	6.8 × 10^−21^
GO:0015630: microtubule cytoskeleton	26	97	3.2 × 10^−9^	3.4 × 10^−8^
GO:0045211: postsynaptic membrane	26	170	0.0002	0.0009
GO:0005643: nuclear pore	25	58	4.1 × 10^−14^	7.7 × 10^−13^
GO:0045121: membrane raft	25	128	4.3 × 10^−6^	2.5 × 10^−5^
GO:0009897: external side of plasma membrane	25	155	0.0001	0.0005
GO:0008021: synaptic vesicle	24	70	4.7 × 10^−11^	5.7 × 10^−10^
GO:0005777: peroxisome	24	105	3.4 × 10^−7^	2.6 × 10^−11^
GO:0014069: postsynaptic density	23	107	1.9 × 10^−6^	1.2 × 10^−5^
GO:0005793: endoplasmic reticulum-Golgi intermediate compartment	22	46	9.8 × 10^−14^	1.7 × 10^−12^
GO:0005819: spindle	22	113	1.7 × 10^−5^	8.9 × 10^−5^
GO:0005765: lysosomal membrane	22	123	6.4 × 10^−5^	0.0003
GO:0010008: endosome membrane	22	145	0.0007	0.003
GO:0001725: stress fiber	21	45	6.6 × 10^−13^	1.0 × 10^−11^
GO:0030426: growth cone	21	72	2.0 × 10^−8^	1.9 × 10^−7^
GO:0043005: neuron projection	21	81	1.9 × 10^−7^	1.6 × 10^−6^
GO:0005905: coated pit	20	46	1.2 × 10^−11^	1.5 × 10^−10^
GO:0031901: early endosome membrane	20	63	8.7 × 10^−7^	9.07 × 10^−8^
GO:0001726: ruffle	20	72	1.1 × 10^−7^	9.2 × 10^−7^
GO:0030659: cytoplasmic vesicle membrane	20	83	1.3 × 10^−6^	8.9 × 10^−6^
GO:0030027: lamellipodium	20	101	3.0 × 10^−5^	0.0002

Cellular compartments identified by at least 20 proteins are included.

Proteins: number of proteins identified in cellular compartment.

NGR: number of annotated proteins in the reference list.

Hyp: hypergeometric *p* value.

Hyp^∗^: corrected hypergeometric *p* value.
